# Blood–brain barrier integrity is linked to cognitive function, but not to cerebral arterial pulsatility, among elderly

**DOI:** 10.1038/s41598-024-65944-y

**Published:** 2024-07-03

**Authors:** Tomas Vikner, Anders Garpebring, Cecilia Björnfot, Lars Nyberg, Jan Malm, Anders Eklund, Anders Wåhlin

**Affiliations:** 1 Department of Diagnostics and Intervention, Umeå University, 90187 Umeå, Sweden; 2 Department of Applied Physics and Electronics, Umeå University, 90187 Umeå, Sweden; 3 Umeå Center for Functional Brain Imaging (UFBI), Umeå University, 90187 Umeå, Sweden; 4 Department of Medical and Translational Biology, Umeå University, 90187 Umeå, Sweden; 5 Department of Clinical Science, Neurosciences, Umeå University, 90187 Umeå, Sweden; 6 Department of Medical Physics, School of Medicine and Public Health, University of Wisconsin-Madison, Madison, WI 53792 USA

**Keywords:** Blood-brain barrier, Blood flow

## Abstract

Blood–brain barrier (BBB) disruption may contribute to cognitive decline, but questions remain whether this association is more pronounced for certain brain regions, such as the hippocampus, or represents a whole-brain mechanism. Further, whether human BBB leakage is triggered by excessive vascular pulsatility, as suggested by animal studies, remains unknown. In a prospective cohort (N = 50; 68–84 years), we used contrast-enhanced MRI to estimate the permeability-surface area product (PS) and fractional plasma volume (${v}_{p}$), and 4D flow MRI to assess cerebral arterial pulsatility. Cognition was assessed by the Montreal Cognitive Assessment (MoCA) score. We hypothesized that high PS would be associated with high arterial pulsatility, and that links to cognition would be specific to hippocampal PS. For 15 brain regions, PS ranged from 0.38 to 0.85 (·10^−3^ min^−1^) and ${v}_{p}$ from 0.79 to 1.78%. Cognition was related to PS (·10^−3^ min^−1^) in hippocampus (β = – 2.9; *p* = 0.006), basal ganglia (β = – 2.3; *p* = 0.04), white matter (β = – 2.6; *p* = 0.04), whole-brain (β = – 2.7; *p* = 0.04) and borderline-related for cortex (β = – 2.7; *p* = 0.076). Pulsatility was unrelated to PS for all regions (*p* > 0.19). Our findings suggest PS–cognition links mainly reflect a whole-brain phenomenon with only slightly more pronounced links for the hippocampus, and provide no evidence of excessive pulsatility as a trigger of BBB disruption.

## Introduction

The blood–brain barrier (BBB) is increasingly studied to understand subtle microvascular damage associated with aging and dementia^1^. The BBB constitutes a semipermeable membrane of endothelial cells, mural cells (smooth muscle cells and capillary pericytes), and astrocytes that protects brain tissue by assuring that unwanted pathological substances and large molecules are retained within the bloodstream^2^. In addition to its barrier function, the BBB contributes substantially to clearance of metabolic waste^2^. Hence, BBB breakdown, i.e., excessive increases in BBB permeability, could initiate a cascade of negative events in the brain.

Dynamic contrast-enhanced (DCE) MRI, utilizing the longitudinal relaxation time (T1) shortening effects of gadolinium, may provide regional estimates of the permeability-surface area product (PS) or ${K}_{trans}$, describing the BBB leakage rate, and of the fractional plasma volume (${v}_{p}$), linked to capillary density^1^. Studies relying on CSF to serum albumin ratios suggest that there is an age-related increase in permeability; however, evidence from imaging studies is scarce and show conflicting results^3^. While some DCE studies support an age-related increase in permeability^4,5^, others found no association^6,7^. DCE studies have also linked BBB leakage to cognitive impairment and dementia. Hippocampal PS correlates with cerebrospinal fluid (CSF) biomarkers of pericyte damage^5^ and is elevated in Alzheimer’s disease (AD) independent of CSF amyloid-$\upbeta$ and tau^8,9^. However, some studies found no associations between hippocampal PS and cognition^10–12^. Others have linked whole-brain gray matter (GM) and white matter (WM) leakages to cognitive decline^13^ and found global PS increases in AD^14^. Hence, whether PS is linked to cognition, and whether such effects are more pronounced for specific brain regions such as the hippocampus, remain largely open questions.

Causal mechanisms underlying capillary damage are not entirely understood^15,16^. Animal studies suggest that BBB disruption could follow as a consequence of altered hemodynamics, such as excessive age-related increases in cardiac-related pulsatility and hypoperfusion^17,18^. However, whether the increases in pulsatility that can be observed in the aging population can cause BBB disruption, or whether the effects of experimentally induced increases in pulse pressure caused by a surgical intervention (e.g., almost 200%^17^) are exaggerated, prompts investigation in human populations. Cerebral arterial hemodynamics are typically assessed with phase contrast (PC) MRI or ultrasound Doppler. Time-resolved 3D PC with 3-directional velocity encoding, or 4D flow MRI, provides whole-brain velocities over the cardiac cycle from a single scan^19,20^. Using a centerline-approach^21^, flow rates^21^ and pulsatility^22,23^ can be derived for the entire cerebral arterial tree. Hence, combining DCE MRI and 4D flow MRI provides an opportunity to inform on the potential relationships between cerebral arterial hemodynamics and microvascular damage. Further, a common challenge in DCE studies is to reliably assess a vascular input function^24^ (VIF), something that could be mitigated by using centerline-methods inspired by 4D flow post-processing tools^21^.

The purpose of this study was to investigate BBB leakages in relation to cognitive function and cerebral hemodynamics among elderly. Specifically, we evaluate PS in multiple brain regions in relation to the Montreal Cognitive Assessment (MoCA) score^25^, to test the hypothesis that hippocampal BBB leakage would show particularly strong links to cognitive function, and in relation cerebral arterial pulsatility, to investigate whether excessive cardiac-related pulsatility transmitted to the microcirculation could have the potential to trigger BBB disruption in humans.

## Materials and methods

### Participants

The initial study population consisted of N = 61 elderly individuals (68 to 84 years). Invitations were sent to individuals of 65 to 85 years, randomly selected from the population registry in Umeå, Sweden. Exclusion criteria included compromised kidney function and allergic reactions to medical treatment, or any other contraindication for intravenous contrast. Eleven participants were excluded from the initial study population; two pilots due to problems with T1 maps; three participants due to problems with the contrast injection (< 1/3 of the cohort-average concentration in blood); one participant did not complete MRI; in one participant there were problems with the 4D flow cardiac gating; four participants due to a history of stroke, something that could bias links between BBB leakage, cognition, and hemodynamics. Hence, the final population consisted of N = 50 (21 women) participants of 68 to 84 years. The experiments were conducted in accordance with the Declaration of Helsinki and approved by the Swedish Ethical Review Authority (dnr: 2020-03710). Written informed consent was provided from all participants prior to the experiments. The anatomical T1-weighted scans were inspected by a neuroradiologist for potential pathological findings. All data were anonymized before any analysis.

### Health assessment

Before the MRI scans, all participants also underwent a medical examination. The examination included a questionnaire where the participants were asked about various medical conditions and medications that were relevant for the study. In addition, participants underwent a MoCA test^25^, where all participants scored at least 20/30, and had their blood pressure measured in the left arm in seated position. Finally, a blood test for creatinine levels was used to exclude participants with renal dysfunction. Descriptive characteristics of the cohort, including results from the health assessment, are summarized in Table 1.

**Table 1:** Descriptive characteristics of the cohort (N = 50).

Health parameter	Mean (SD)
Age (years)	76.3 (4.1)
Sex (men/women)	31/19
Body mass index	25.1 (3.7)
Diabetes mellitus	6
Hypertension	29
Hyperlipidemia	25
Atrial fibrillation	9
Transient ischemic attack	2
SBP (mmHg)	135 (17)
DBP (mmHg)	75.3 (9.3)
MoCA score	26.3 (2.2)
ICA PI	1.14 (0.20)
Distal arterial PI	0.99 (0.16)
tCBF (ml/min)	556 (98)

*Participants with both DCE MRI and 4D flow MRI. The internal carotid artery (ICA) pulsatility index (PI) was obtained by averaging left and right ICA waveforms. Total cerebral blood flow (tCBF) refers to the sum of the inflow through the ICAs and the basilar artery. SD, standard deviation.

### Magnetic resonance imaging

All MRI scans were done with a 3 Tesla scanner (Discovery MR 750; GE Healthcare, Milwaukee, Wisconsin) with a 32-channel head coil. These included 4D flow MRI for arterial blood flow and pulsatility, structural T1 weighted scans for brain segmentation and as a basis for co-registration, and DCE MRI and T1 mapping to assess microvascular integrity (PS and ${v}_{p}$).

*4D flow MRI.* The 4D flow scans were obtained with a radial PC-VIPR (phase-contrast vastly undersampled isotropic-voxel projection reconstruction) sequence, providing flow velocities in all spatial directions (x, y, z), time-resolved over one averaged cardiac cycle, with 3D coverage of the entire brain^19^. The 4D flow scans were obtained with the following parameters: 5-point velocity encoding^20^ (venc): 110 cm/s, repetition time/echo time (TR/TE) 6.5/2.7 ms, flip angle 8$^\circ$, radial projections 16,000, and imaging volume 22 × 22 × 22 cm^3^. Total scan time was approximately 9 min. Magnitude, velocity, and complex (CD) data were reconstructed into 20 frames per cardiac cycle and an isotropic voxel size of 0.6875 mm, using parallel imaging and gridding with localized sensitivities (PILS)^26^. Polynomial fitting to the background field was used to correct for gradient errors caused by eddy currents and concomitant fields^27^.

*T1 weighted MRI.* A 3D fast-spoiled gradient echo (FSPGR) sequence was used for the T1 weighted scans, using the following parameters: TR/TE 8.2/3.2 ms, flip angle 12$^\circ$, 176 slices with 1 mm thickness, in-plane resolution 0.94 mm, field of view 25 × 25 cm^2^ and a phase acceleration of 2.

*Dynamic contrast enhanced MRI.* The DCE MRI scans included a series (60 time frames) of T1 weighted 3D FSPGR acquired over 17.5 min (temporal resolution 17.5 s) through an axial slab with slab-selective excitation. Intravenous contrast (0.1 mmol/kg Dotarem) was administered after about one minute using a slow injection, lasting approximately one minute. Imaging parameters: TR/TE: 4.5/1.6 ms, flip angle 12$^\circ$, slice thickness 1.5 mm, in-plane resolution 0.82 mm, field of view 21 × 21 cm^2^ over 108 slices, partial Fourier 6/8, and parallel imaging factor 2.

*T1 mapping.* The T1 map was obtained using the variable flip angle (VFA) method^28^. Before the DCE session, a set of three T1 weighted FSPGR scans with the same imaging parameters as the DCE sequence, but flip angles 2, 8, and 12 degrees, were acquired. Two frames (17.5 s per frame) were collected (and averaged) for each flip angle.

*B1 mapping.* Before T1 mapping, a B1 map was acquired using the Bloch-Siegert shift approach^29^ to correct for deviations and spatial inhomogeneities in the achieved compared to the intended flip angle, using the following imaging parameters: flip angle 15$^\circ$, slice thickness 5 mm, and in-plane resolution 3.91 mm.

### Region of interest extraction

Statistical parametric mapping (SPM12) was used to segment GM and WM tissue probability maps. These maps were then thresholded to include either GM or WM probabilities > 0.95 to minimize influence of CSF boundaries near tissue. FreeSurfer 6.0 was used to segment regions of interest^30^ (ROIs). Cortical and WM subregions were combined into frontal, parietal, temporal, occipital and cingulate GM and WM regions^31^, and further combined over the left and right hemispheres. Only normal-appearing white matter, free from white matter lesions, were included in the WM ROIs. Thalamus, caudate, putamen, pallidum, and hippocampus were used as subcortical GM ROIs, also combined over the hemispheres. Regional brain volumes were obtained from FreeSurfer and normalized by intracranial volume as described previously^32^.

### DCE MRI processing

The T1 weighted DCE series and scans acquired for T1 mapping (flip angles 2, 8, and 12 degrees) were initially pre-processed in MATLAB R2022b (Natick, Massachusetts: The MathWorks Inc) with a filter to reduce the influence of Gibbs ringing artifacts^33^. Next, all volumes were co-registered to the high-resolution T1 weighted volume using an initial rigid-body co-registration followed by a boundary-based co-registration^34^ (using FreeSurfer 6.0).

The rest of the DCE MRI and T1 mapping analyses were performed in MATLAB. The VFA method^28^ was used to calculate T1 maps from the T1 weighted scans acquired with three flip angles (2, 8, and 12 degrees). The B1 corrected flip angles (nominal flip angle multiplied by B1 map) was used as input for the VFA method and for deriving Gadolinium (Dotarem) concentration curves. The concentration curves were estimated from the baseline T1 maps (${T}_{10}$) and the DCE MRI time curves (assuming fast water exchange rates^35^) according to:

$$C(t)=\frac{1}{{r}_{1}}\left({R}_{1}\left(t\right)-{R}_{10}\right),$$

where ${r}_{1}$ is the relaxivity of the contrast agent (3.5 L$\cdot$mmol^−1^ s^−1^ in blood plasma^36^), ${R}_{10}=\frac{1}{{T}_{10}}$ is the relaxation rate at baseline, and ${R}_{1}\left(t\right)$ is the relaxation rate as a function of time (or concentration), estimated from the DCE time curves and T1 maps as described in detail elsewhere^35^.

The superior sagittal sinus (SSS) was selected for vascular input function (VIF) extraction, as that vascular region is less sensitive to motion artifacts, partial volume errors, and inflow effects^1^. Seed points were manually placed in the posterior SSS by visual inspection of the DCE data at the time point of maximum signal enhancement. Then, an SSS centerline was obtained by cross-correlating all DCE voxel time curves with the seed voxel time curve, and applying a strict correlation threshold of R = 0.99, followed by centerline extraction by an algorithm commonly used in 4D flow MRI post-processing^21^. The SSS curves were then averaged along 25 centerline voxels to obtain the final VIF plasma concentration (${C}_{VIF}$), defined as:

$${C}_{VIF}\left(t\right)=\frac{{C}_{b}\left(t\right)}{1-HCT},$$

where a ${C}_{b}(t)$ is the Gadolinium concentration in blood and a hematocrit (HCT) of 0.45 was assumed^37^. The VIF extraction was performed by two raters to evaluate inter-rater reliability.

Finally, ROI-averaged tissue concentration curves (${C}_{tissue}$) were obtained by averaging the concentration over all voxels within each ROI (using no thresholding or exclusion based on extreme values). The Patlak model was used to estimate PS and ${v}_{p}$, assuming unidirectional (irreversible) flow from the BBB to the extracellular fluid^1,38^. The tracer concentration ${C}_{tissue}$ was then modelled as a function of ${C}_{VIF}$, PS and ${v}_{p}$, according to the Patlak equation, which was solved for PS and ${v}_{p}$ using multiple linear regression:

$${C}_{tissue}\left(t\right)=PS{\int }_{0}^{t}{C}_{VIF}\left(\tau \right)d\tau +{v}_{p}{C}_{VIF}(t).$$

**Figure 1:**
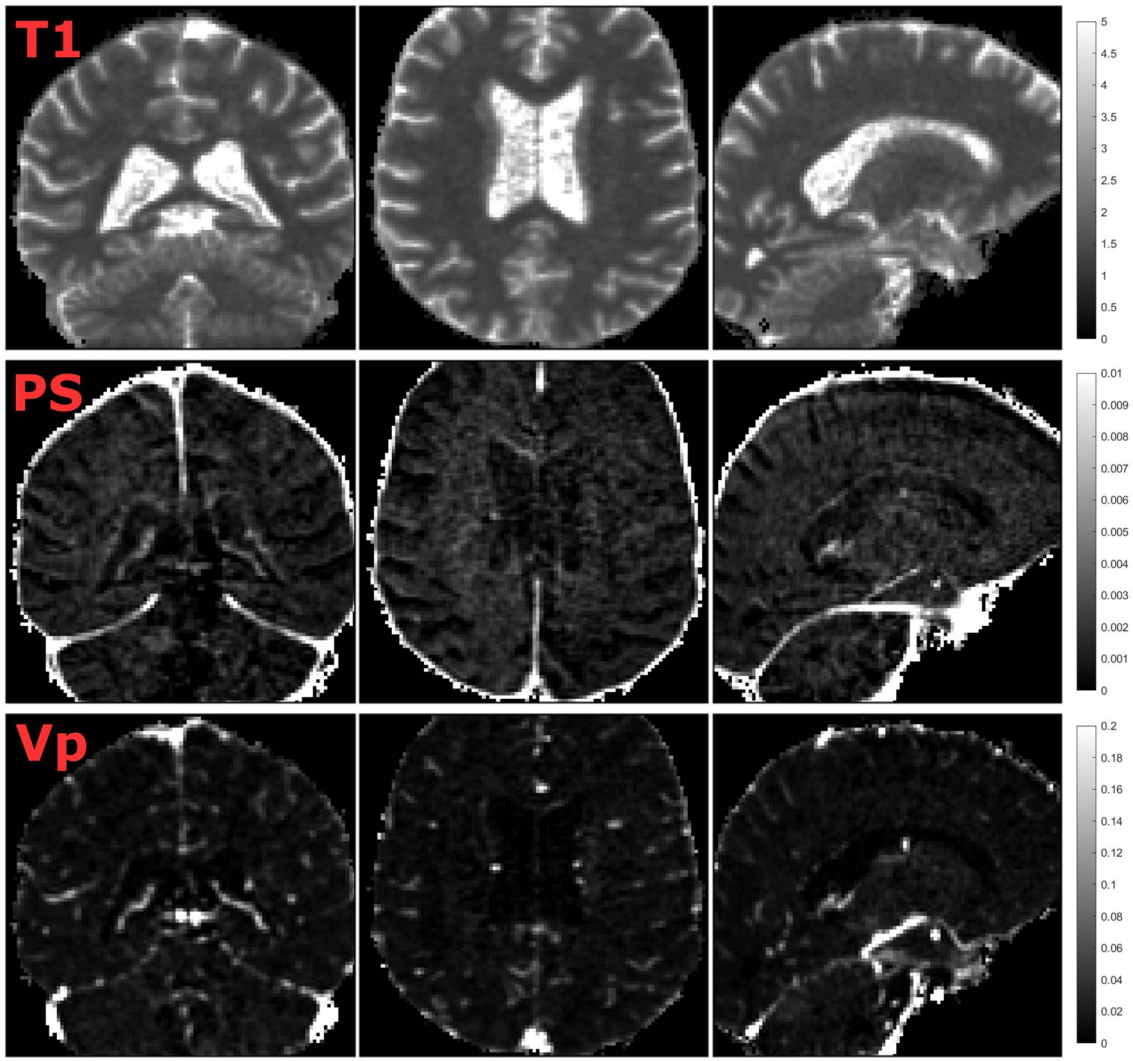
T1 maps (seconds) at baseline, the permeability-surface area product (PS; min^−1^), and fractional plasma volume (${v}_{p}$) for an example participant. The T1 maps were obtained using the variable flip angle method, and PS and ${v}_{p}$ were obtained using Patlak analysis. Note that while the images are thresholded according to the color bars to aid visualization, no thresholding or exclusion based on extreme values was used for any of the results.

This was done on a voxel-level for visualization (Fig. 1) and on a ROI-level for evaluation of PS and ${v}_{p}$ against other variables (e.g., age, cognition, and 4D flow hemodynamics). In addition, PS and ${v}_{p}$ were averaged over basal ganglia, WM, cortical GM, and whole-brain (15 × ROIs) for evaluation against MoCA and 4D flow hemodynamics. The full ${C}_{tissue}$ and ${C}_{VIF}$ curves were used as input to the Patlak model for the main analyses. Since the contrast peak may not always be accurately estimated due to limited temporal resolution of the DCE scan, supplementary analyses were performed to assess the effect of including vs. excluding the contrast injection peak (Sup. Fig. 1). Further, as the RF pulse excitation profile may be non-uniform at the edges of the axial slab (Sup. Fig. 3), supplementary analyses between region-averaged PS values and MoCA score were performed after excluding the frontal and parietal cortex (Sup. Fig. 4).

### 4D flow MRI processing

**Figure 2:**
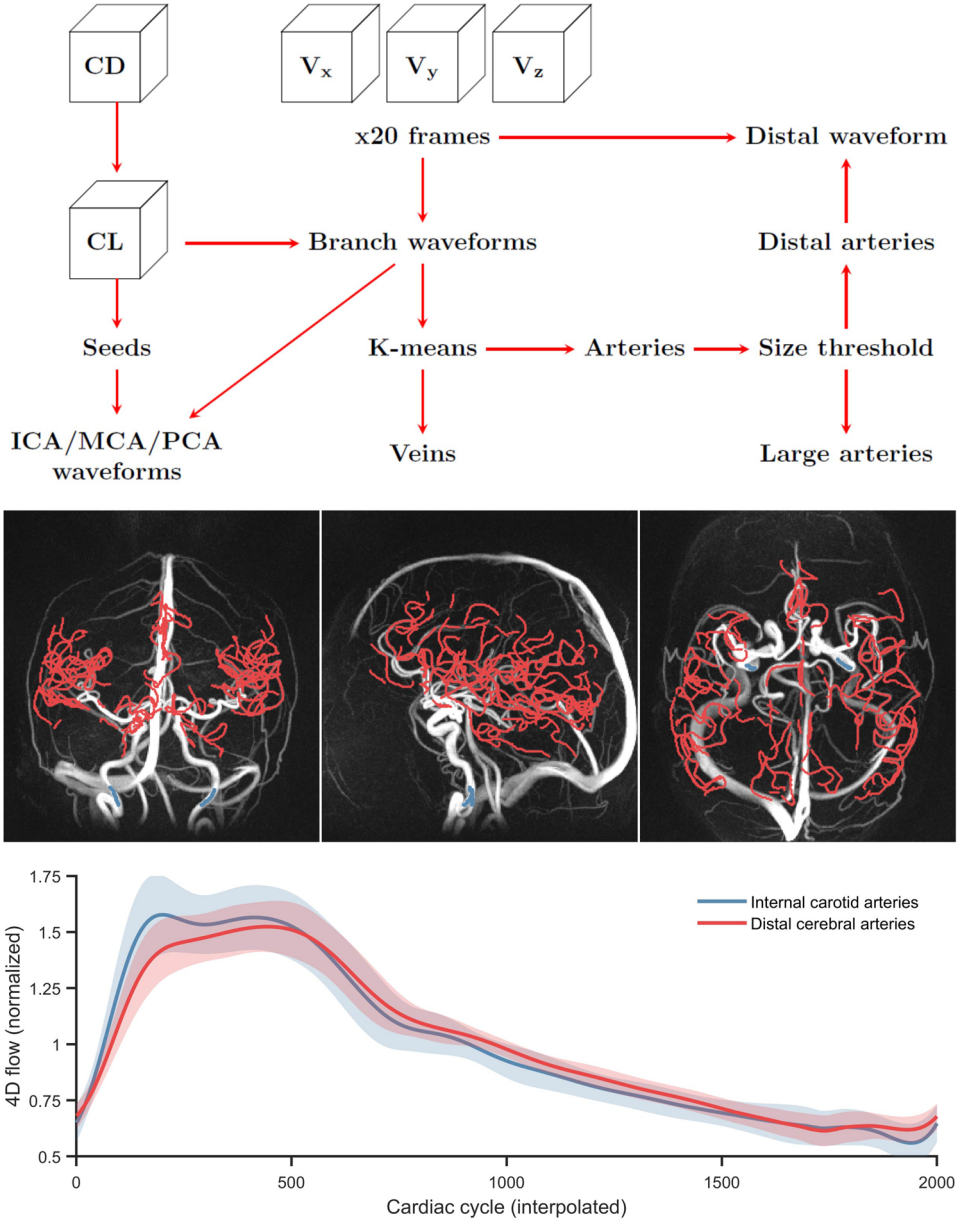
4D flow MRI complex difference (CD) maximum intensity projections and centerlines (CLs) used to assess distal arterial (red) and internal carotid artery (ICA; blue) pulsatility. The waveforms represent group-averaged ICA and distal flows after interpolation and normalization (division by the means). The schematic shows how the CD, CLs, and velocities (${\text{V}}_{x}$, ${\text{V}}_{y}$, ${\text{V}}_{\text{z}}$) were used for automatic distal arterial waveform extraction and manual seed-point based waveform extraction in ICA and middle/posterior cerebral arteries (MCA/PCA).

All 4D flow data were processed in MATLAB R2022b. Flow waveforms, time-resolved over the cardiac cycle, were estimated for the left and right internal carotid arteries (ICA), middle cerebral arteries (MCA), posterior cerebral arteries (PCA), the basilar artery, and for distal cerebral arteries using semi-automatic post-processing schemes that utilize centerline representations of the cerebral vascular tree^21,23^. In short, intensity-based thresholding of the CD angiogram was first applied to obtain a binary representation of the vascular tree. Then, a skeletonization algorithm was used to automatically prune the binary representation into a one voxel thick skeleton. The vascular skeleton was then subdivided into centerline branches at each junction point, with no spacing between centerline points (Fig. 2). For all voxels in the centerline structure, flow direction was automatically estimated by considering the position of the voxel as well as 6/4 neighboring voxels (3 in each direction for large arteries, 2 for distal arteries). Finally, a local intensity-based threshold was used to separate the vessel cross section from background tissue.

For ICA, MCA, PCA and BA segments, seed points were manually placed at the cervical ICA, MCA M1, and PCA P2, and waveforms were sampled and averaged along 15 neighboring cross-sections. The left and right ICA waveforms were averaged to obtain a single ICA waveform. However, the individual (left and right) ICA, MCA, and PCA waveforms were also analyzed separately in relation to PS and ${v}_{p}$ in the brain regions they supply. To identify distal arteries, a hessian-based vessel enhancement filter^40^ was applied to the CD volume to enhance visualization of small vessels, and branch-averaged flow waveforms were obtained for all segmented centerline branches. Distal arteries were then automatically segmented by using K-means clustering (K = 2) to separate arteries of veins (using branch-averaged waveforms as input) followed by a diameter threshold of 1.25 mm to separate large arteries from small (i.e., distal) arteries (Fig. 2). This post-processing method for distal arterial pulsatility has been described in detail previousy^23^. The automatically segmented distal arterial tree was also manually pruned to minimize contamination from veins and extracranial vessels. Finally, waveforms along the centerline voxels were normalized, and a final waveform representation was estimated as a median of the normalized waveforms. For one individual, the automatic segmentation of distal cerebral arteries failed (due to noisy data), and a manual segmentation was used instead. The manual segmentation was obtained by pruning the full centerline tree to contain the anterior, middle, and posterior cerebral arteries, to include the A2, M2, and P2 segments and all distal connecting branches.

All flow waveforms were interpolated from 20 to 2000 frames per cardiac cycle using cubic splines (Fig. 2) before pulsatility analysis. The pulsatility index (PI), defined as the waveform amplitude divided by the mean flow rate over the cardiac cycle, was used to assess pulsatility. Total cerebral blood flow (tCBF) was defined as the sum of the inflow through the ICAs and the BA.

### Statistics

All statistical analyses were performed in MATLAB R2022b. Skewness and kurtosis were used to assess normality of PS and ${v}_{p}$. Intraclass correlation (ICC) was used to assess interrater reliability of the VIF extraction and to evaluate exclusion of early frames before Patlak fit. Linear (Pearson) correlation (R) and regression (β) were used to evaluate the associations among PS, ${v}_{p}$, age, MoCA, tCBF, and PI. For all statistical tests, *p* < 0.05 was considered significant. No adjustments were made for multiple comparisons. Regression (Residualization) was used to adjust the variables of interest for covariates, by fitting a regression model with the covariates as predictors and the variable of interest as dependent variable, and keeping the residuals as the adjusted variable. This approach was used to correct PS–MoCA associations for age and sex in the main results (Fig. 4), and to correct PS for ${v}_{p}$ and regional brain volumes for intracranial volume in supplementary tables (Sup. Table 2–3). Principal component analysis was used to assess shared variance in PS and ${v}_{p}$.

**Table 2:** Permeability-surface area product (PS) and fractional plasma volume (${v}_{p}$) descriptive values for cerebral gray and white matter (WM) regions and associations and PS and ${v}_{p}$ within each region.

	PS (10^−3^ min^−1^)		${v}_{p}$(%)		PS-${v}_{p}$
	Mean	95% CI	Mean	95% CI	R (p)
Cortex					
Frontal	0.52	0.46, 0.57	1.18	1.13, 1.23	0.19 (0.18)
Parietal	0.52	0.46, 0.58	1.22	1.17, 1.27	0.28 *
Temporal	0.72	0.67, 0.78	1.32	1.26, 1.38	0.40 **
Occipital	0.86	0.77, 0.96	1.73	1.63, 1.84	0.53 ***
Cingulate	0.56	0.49, 0.64	1.49	1.41, 1.57	0.51 ***
White matter					
Frontal	0.52	0.44, 0.60	0.81	0.77, 0.85	0.41 **
Parietal	0.48	0.40, 0.55	0.79	0.75, 0.83	0.32 *
Temporal	0.49	0.42, 0.57	0.82	0.78, 0.87	0.41 **
Occipital	0.39	0.31, 0.48	0.80	0.76, 0.84	0.34 *
Cingulate	0.58	0.49, 0.68	0.91	0.86, 0.97	0.51 ***
Subcortical					
Thalamus	0.54	0.45, 0.63	1.68	1.60, 1.76	0.49 ***
Caudate	0.45	0.36, 0.53	1.47	1.40, 1.54	0.39 **
Putamen	0.66	0.58, 0.72	1.65	1.58, 1.72	0.24 (0.09)
Pallidum	0.63	0.53, 0.73	1.20	1.13, 1.26	0.40 **
Hippocampus	0.64	0.56, 0.73	1.78	1.67, 1.89	0.35 *

95% confidence intervals (CI) of the cohort mean (N = 54). R and *p*-values obtained from Pearson correlation. Unadjusted *p*-values: *p < 0.05; **p < 0.01; ***p < 0.001. WM ROIs include normal-appearing WM free from WM lesions.

**Table 3:** Permeability-surface area product (PS) and fractional plasma volume (${v}_{p}$) for cerebral gray and white matter (WM) regions in relation to age and cognition.

	Age—PS (10^−3^ min^−1^)	Age—${v}_{p}$ (%)	PS (10^−3^ min^−1^)—MoCA	${v}_{p}$(%)—MoCA
	β (CI)	β (CI)	β (CI)	β (CI)
Cortex				
Frontal	− 0.01 (− 0.02, 0.004)	0.00 (− 0.02, 0.006)	− 3.1 (− 6.3, 0.1)	− 0.9 (− 4.8, 2.9)
Parietal	0.00 (− 0.02, 0.01)	0.00 (− 0.02, 0.01)	− 2.3 (− 5.3, 0.7)	0.7 (− 2.8, 4.3)
Temporal	− 0.01 (− 0.02, 0.01)	− 0.01 (− 0.02, 0.01)	− 3.2 (− 6.2, − 0.2) *	0.33 (− 2.7, 3.4)
Occipital	− 0.01 (− 0.03, 0.01)	− 0.02 (− 0.04, 0.01)	− 1.7 (− 3.6, 0.2)	− 0.2 (− 2.0, 1.5)
Cingulate	− 0.02 (− 0.04, 0.002)	− 0.02 (− 0.04, − 0.01) *	− 0.6 (− 3.0, 1.7)	− 0.7 (− 3.0, 1.5)
White matter				
Frontal	− 0.01 (− 0.03, 0.004)	− 0.01 (− 0.02, 0.002)	− 2.4 (− 4.7, − 0.2) *	− 3.2 (− 7.6, 1.2)
Parietal	0.00 (− 0.02, 0.01)	0.00 (− 0.01, 0.01)	− 2.5 (− 4.9, − 0.2) *	− 1.1 (− 5.7, 3.6)
Temporal	0.00 (− 0.02, 0.01)	0.00 (− 0.01, 0.01)	− 3.1 (− 5.5, − 0.8) *	− 1.1 (− 5.6, 3.5)
Occipital	0.00 (− 0.02, 0.02)	0.01 (− 0.004, 0.01)	− 2.3 (− 4.5, − 0.2) *	− 0.3 (− 5.2, 4.7)
Cingulate	− 0.02 (− 0.04, 0.002)	− 0.02(− 0.03, − 0.005) **	− 0.9 (− 2.8, 1.0)	− 0.6 (− 4.0, 2.8)
Subcortical				
Thalamus	− 0.02 (− 0.37, 0.008)	− 0.02 (− 0.04, − 0.003) *	− 1.1 (− 3.1, 0.9)	0.32 (− 2.1, 2.7)
Caudate	− 0.01 (− 0.04, 0.01)	− 0.01 (− 0.03, − 0.01)	− 1.7 (− 3.8, 0.5)	− 1.2 (− 3.8, 1.4)
Putamen	− 0.01 (− 0.03, 0.01)	− 0.01 (− 0.03, 0.01)	− 3.2 (− 5.5, − 0.9) **	− 1.0 (− 3.6, 1.7)
Pallidum	− 0.02 (− 0.04, 0.003)	− 0.01 (− 0.03, 0.002)	− 1.8 (− 3.6, − 0.01) *	− 0.1 (− 2.9, 2.6)
Hippocampus	− 0.01 (− 0.03, 0.01)	− 0.04 (− 0.06, − 0.01) **	− 2.9 (− 4.9, − 0.9) **	− 0.5 (− 2.2, 1.1)

β-values and confidence intervals (CI) were obtained from linear regression. Cognition was assessed by the Montreal Cognitive Assessment (MoCA) test. Unadjusted *p*-values: *p < 0.05; **p < 0.01. $\upbeta$-values reflect change in PS (10^−3^ min^−1^) per change in age (years) or change in MoCA per change in PS (10^−3^ min^−1^).

## Results

### Regional estimates of BBB leakage rates and fractional plasma volume

**Figure 3:**
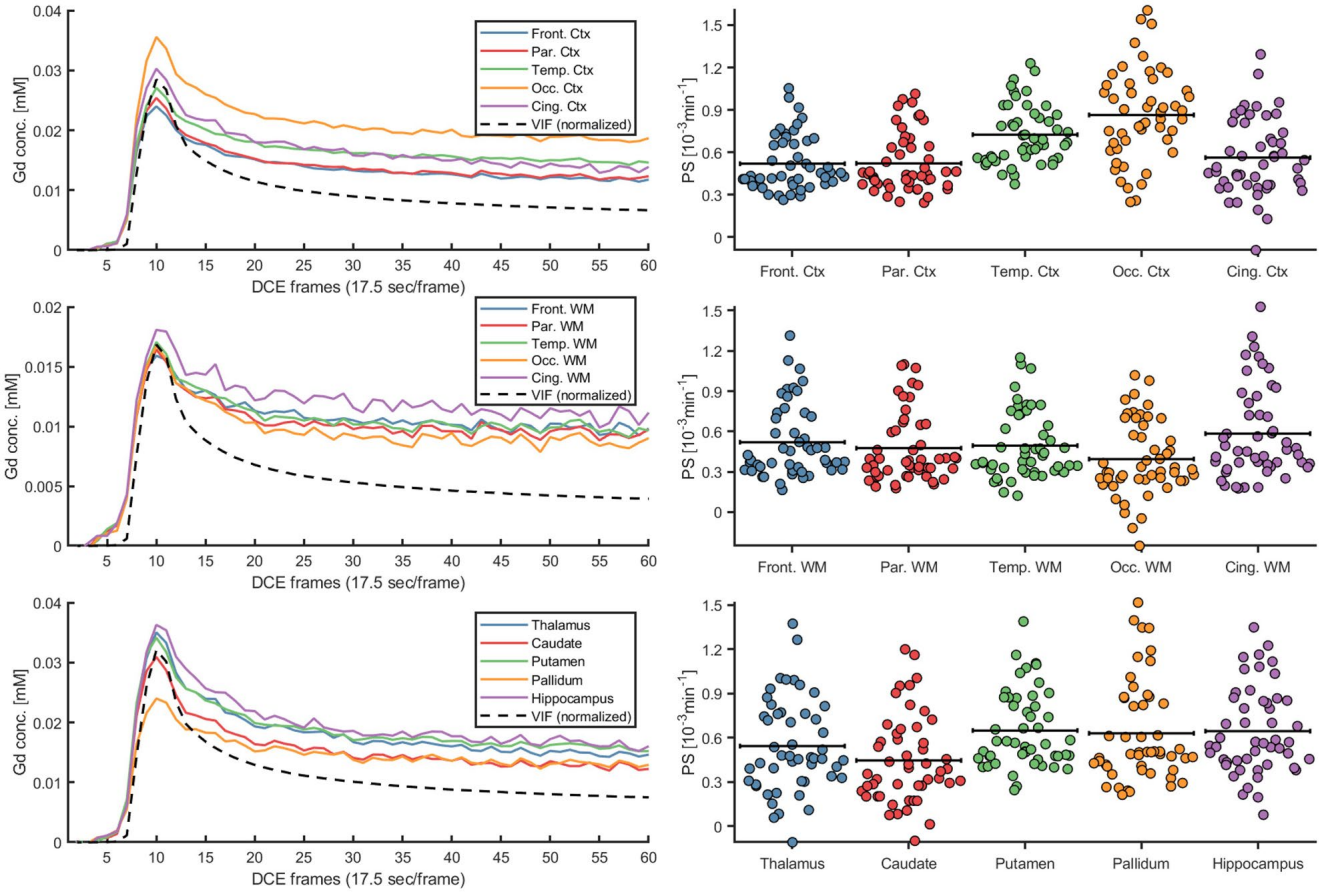
Group-averaged gadolinium (Gd; Dotarem) concentration [mM] for cortical (Ctx), white matter (WM) and subcortical structures. The concentration curves were averaged across the entire region within each subject to obtain individual concentration estimates, and then group-averaged for visualization. The vascular input function (VIF) concentration is normalized to the region-average tissue concentration peak to facilitate visualization together with the tissue concentration curves. The swarm charts represent individual (N = 50) permeability-surface area product (PS) estimates where black lines correspond to the mean value.

The Gadolinium curves showed similar shapes for all ROIs, with higher concentrations in cortical and subcortical GM compared to WM (Fig. 3), as also seen on individual concentration curves (Sup. Fig. 1). Hippocampal and whole-brain PS estimates showed excellent agreement (ICC 0.94–0.96) when comparing the effect of excluding the contrast injection peak from the concentration curves vs. using full concentration curves as input to the Patlak model (Sup. Fig. 2), and results obtained using full concentration curves were used for all subsequent analyses. For all 15 ROIs, ${v}_{p}$ was normally distributed (skewness – 0.28 to 0.90; kurtosis – 0.69 to 0.70) and PS was approximately normally distributed (skewness 0.18–1.08; kurtosis – 0.51 to 0.25), with group-average values between 0.39$\cdot$10^−3^ and 0.86$\cdot$10^−3^ min^−1^ for PS and 0.79% to 1.78% for ${v}_{p}$ (Table 2). The influence of the VIF extraction (in the SSS using a centerline approach) on PS and ${v}_{p}$ was evaluated based on measurements by two raters (T.V. & C.B.). The interrater reliability was excellent, as indicated by an ICC > 0.99 for PS and an ICC > 0.98 for ${v}_{p}$ for all 15 ROIs. Further, since we expected that high PS and ${v}_{p}$ would partially reflect a whole-brain phenomenon, we extracted correlation matrices for all 15 regions and found between-region correlations between r = 0.36 and 0.98 (average 0.74 ± 0.13) for PS and between r = 0.44 and 0.92 for ${v}_{p}$ (average 0.70 ± 0.10). Furthermore, principal component analysis indicated that 76% of the variance in PS and 73% of the variance in ${v}_{p}$ could be explained by the 1st principal component. Finally, considering that PS is the product of permeability and surface area, we also evaluated correlations between PS and ${v}_{p}$ and found moderate correlations for most ROIs (Table 2).

### Associations to cognitive function and age

**Figure 4:**
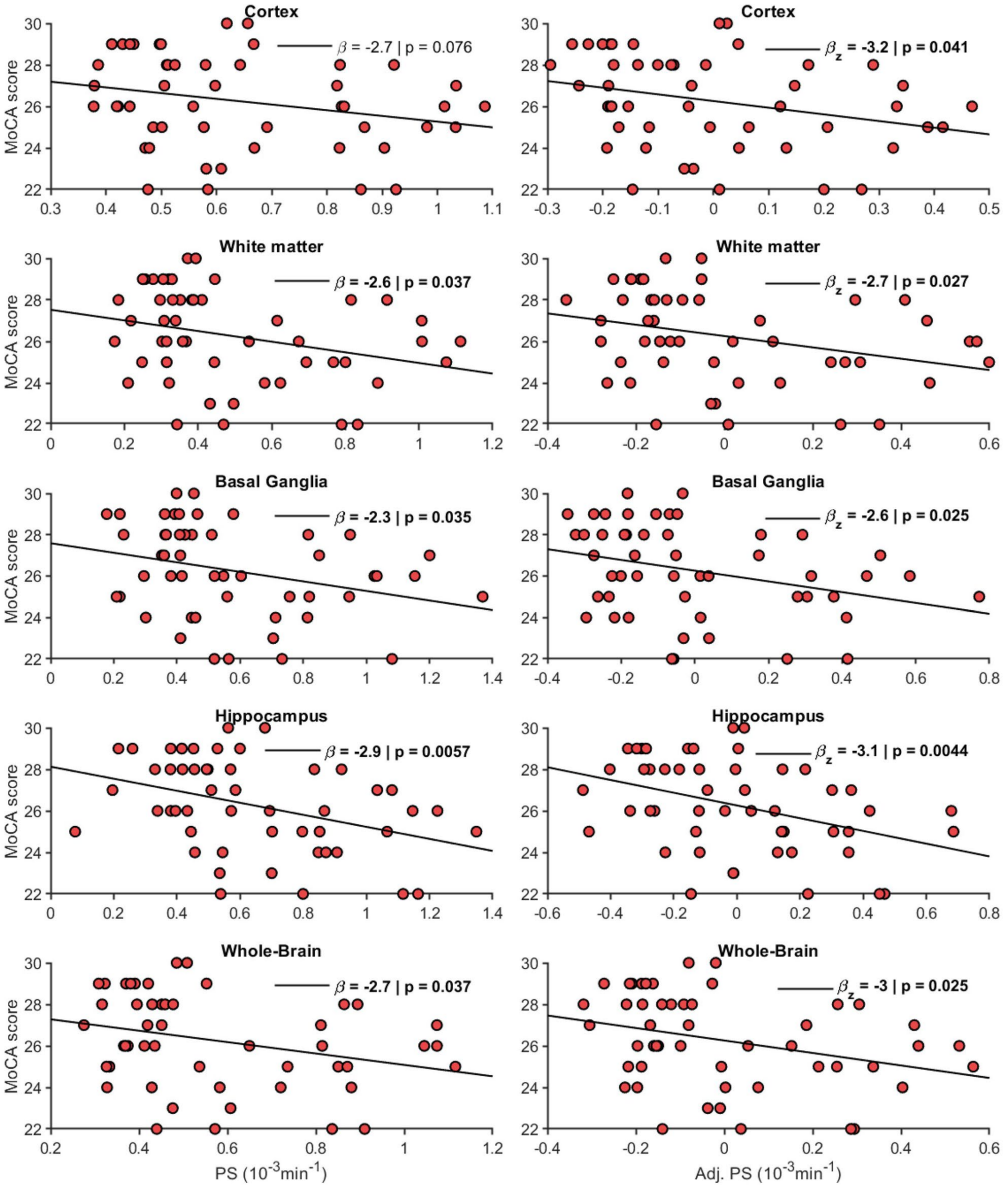
Permeability-surface area product (PS; 10^−3^ min^−1^) in relation to the Montreal cognitive assessment (MoCA) score for cortex and white matter (WM) lobes, average over frontal, parietal, temporal, occipital and cingulate regions, for basal ganglia (BG), averaged over caudate, putamen and pallidum, and for hippocampus and whole-brain, averaged over all 15 regions. The adj. was adjusted for age and sex by residualization. The β coefficients indicate change in MoCA score per 1.0 × 10^−3^ min^−1^ change in PS.

PS was inversely related to cognition assessed as the MoCA score for the temporal cortex, frontal, parietal, temporal and occipital WM, putamen, pallidum and the hippocampus (Table 2). When evaluating hippocampal PS and region-averaged PS values for the cortex, WM and basal ganglia, PS was related to MoCA score for the hippocampus (β = – 2.9 change in MoCA per 1.0 × 10^−3^ min^−1^ change in PS; *p* = 0.006), WM (β = – 2.6; *p* = 0.04), basal ganglia (β = – 2.3; *p* = 0.04), and borderline-related for cortex (β = – 2.7; *p* = 0.08) (Fig. 4). These findings were not suppressed but slightly more pronounced and significant for all regions when adjusting for age and sex (Fig. 4). Further, whole-brain PS was also linked to MoCA in unadjusted (β = – 2.7; *p* = 0.04) and age and sex adjusted (*p* = 0.03) models, and similar results were obtained when excluding early frames before the Patlak fit (Sup. Fig. 2), and when excluding the frontal and parietal cortex to minimize errors due to a non-uniform RF pulse excitation profile near the edges of the axial slab (Sup. Fig. 3–4). Hence, while our findings do support a link between BBB leakage and cognition in old age, they also suggest that this effect is a brain wide mechanism rather than an isolated effect in the hippocampus.

In contrast to previous findings suggesting that hippocampal PS increases with age^5^, we found no age–PS association for the hippocampus or any other region. Instead, we found non-significant negative trends for all regions (Table 3). On the contrary, ${v}_{p}$ was inversely related to age for some regions, including the hippocampus, thalamus, cingulate cortex, and cingulate WM (Table 3), potentially indicating reduced capillary density with increasing age. However, ${v}_{p}$ was unrelated to MoCA score in our cohort (Table 3) and adjusting PS for ${v}_{p}$ had little impact on the PS–MoCA associations (Sup. Table 2).

### Associations to cerebral hemodynamics

tCBF rate was estimated to 556 ± 96 ml/min (ICA + BA) and arterial PI to 1.14 ± 0.19 and 0.99 ± 0.16 for ICA and distal cerebral arteries, respectively (Table 1). The PI was unrelated to both PS and ${v}_{p}$ when comparing whole-brain pulsatility estimates (left/right ICA-averaged PI and distal PI) in relation to PS and ${v}_{p}$ in the hippocampus, and in relation to region-averaged PS and ${v}_{p}$ for the cortex, WM, and basal ganglia, and whole-brain (Table 4). Local associations were also evaluated by considering PI and flow rates at specific vessel segments for the ROIs they supply, while also considering left and right sides separately. Yet, no links were found between ICA PI in relation to frontal cortex, frontal WM and hippocampus, between MCA PI in relation to temporal cortex, temporal WM and basal ganglia, or between PCA PI in relation to occipital cortex, occipital WM and hippocampus (Sup. Table 1).

**Table 4:** Permeability-surface area product (PS) and fractional plasma volume (${v}_{p}$) in relation to 4D flow MRI derived total cerebral blood flow (tCBF) rate and pulsatility index (PI).

Region	tCBF-${v}_{p}$ (%)	tCBF-PS (10^−3^ min^−1^)	DPI-${v}_{p}$ (%)	DPI-PS (10^−3^ min^−1^)
	β (CI)	β (CI)	β (CI)	β (CI)
Cortex	0.93 (0.33, 153)**	0.51 (− 0.08, 1.10)	− 0.01 (− 0.41, 0.40)	0.07 (− 0.30, 0.44)
Cerebral WM	0.60 (0.25, 0.94)**	0.64 (− 0.09, 1.38)	− 0.04 (− 0.27, 0.20)	− 0.02 (− 0.49, 0.45)
Hippocampus	1.52 (0.46, 2.58)**	0.63 (− 0.22, 1.49)	− 0.03 (− 0.73, 0.67)	0.01 (− 0.53, 0.55)
Basal ganglia	1.04 (0.45, 1.63)***	0.64 (− 0.20, 1.47)	− 0.15 (− 0.55, 0.25)	− 0.09 (− 0.62, 0.43)
Whole-brain	0.90 (0.41, 1.40)***	0.60 (− 0.08, 1.30)	− 0.06 (− 0.4, 0.28)	− 0.01 (− 0.45, 0.24)

β-values and confidence intervals (CI) were obtained from linear regression. While DPI reflects distal arterial PI, similar results were obtained for internal carotid PI. The tCBF (L/min) is defined as the sum of the internal carotid and the basilar artery inflow, scaled to L/min to be on similar scale as ${v}_{p}$ (%) and PS (10^−3^ min^−1^). Unadjusted *p*-values: *p < 0.05; **p < 0.01; ***p < 0.001.

Regarding blood flow rates, tCBF and ${v}_{p}$ were moderately correlated for all ROIs (Table 4), and local relationships were also observed between ICA flow rate and ${v}_{p}$ in frontal cortex, frontal WM and hippocampus for the left hemisphere, and between MCA flow rate and ${v}_{p}$ in temporal cortex, temporal WM, and basal ganglia for both hemispheres (Sup. Table 1). Weak positive trends were also found between tCBF and PS (Table 4); however, adjusting PS for ${v}_{p}$ also suppressed these trends (Sup. Table 2).

### Regional brain volumes

Finally, regional brain volumes were evaluated in relation to age, PS, distal PI and MoCA score. This revealed an inverse relationship between age and regional brain volumes for 7/15 brain regions, including the temporal and occipital cortex, frontal, temporal and occipital WM, thalamus and hippocampus (Sup. Table 3). In contrast to previous findings of inverse relationships between PI and regional brain volumes^41^, distal PI was positively related to frontal WM volume (*p* = 0.015); however, this single association on 15 tests would not survive a multiple comparisons correction. Regional volumes were unrelated to PS and MoCA for all 15 investigated ROIs.

## Discussion

We estimated BBB leakage rates (PS) and fractional plasma volume (${v}_{p}$) for several cortical, WM, and subcortical GM regions using DCE MRI. While we observed large interregional differences in BBB leakage rates, there were also pronounced interregional correlations (i.e., a strong global mode of interindividual differences), as suggested by the high amount of variance explained by the 1^st^ principal component. Further, while hippocampal PS showed a moderate inverse relation to cognition based on the MoCA score, similar associations were also found for white matter and the basal ganglia, as well as borderline-significant associations for cortex. Finally, we tested the hypothesis that excessive arterial pulsatility, transmitted to the cerebral microcirculation, would trigger BBB disruption^17^. However, 4D flow MRI derived cerebral arterial pulsatility was unrelated to PS, suggesting that BBB disruption occurs via a different pathway.

Previous DCE studies on subtle BBB leakages report widely different values, sometimes differing by several orders of magnitude^42^. However, our average PS values of 0.39$\cdot$10^−3^ min^−1^ to 0.86$\cdot$10^−3^ min^−1^ over a 3D volume covering the entire brain are relatively similar to those reported in a recent study of cognitively healthy elderly individuals (54 to 76 years) describing regional PS differences in a coronal 2D slice of the brain^7^. Other studies report widely different values, both within the spectrum of normal aging and dementia^42^. This could partially be attributed to the use of different Gadolinium based contrast agents, but also due to differences in acquisition and post-processing methods. Further, our ${v}_{p}$ estimates ranged from 0.79 to 1.78% for the 15 ROIs, suggesting that there are pronounced differences in capillary density and/or lumen size across the brain. Note that providing actual values of microvascular volume fraction would require knowledge about venous to capillary hematocrit ratios. Fractional plasma volume reference values are typically not reported in DCE MRI studies of subtle microvascular damage. However, our observed cortical (1.29%) and WM (0.81%) averages suggest ~ 37% lower ${v}_{p}$ in WM comparted to the cortex, aligning with a large histopathological study where WM microvascular densities were 20 – 49% lower than in the overlaying cortex^43^.

In contrast to our findings, Montagne et al.^5^ found pronounced PS–age associations for the hippocampus, although their sample involved a broad age span (23 to 91 years), and Verheggen et al.^4^ found an age-relation for whole-brain GM and WM in healthy individuals (47 to 91 years). However, in line with our findings, Ha et al.^7^ found no age-relation in a healthy sample (age 54 to 76 years), Taheri et al.^6^ found no age-relation in a mixed sample (age 22 to ~ 90 years), and Nation et al.^8^ found no age-relation (age 45 to ~ 90 years) in the full cohort or when subdividing groups based on cognitively healthy and cognitively impaired. Hence, while there appears to be some BBB degeneration with aging, evidence from imaging studies is inconsistent. Importantly, Montagne et al.^5,9^ and Nation et al.^8^ linked hippocampal BBB permeability to MCI^5^ and AD^8^, and to cognitive decline in ApoE4 + carriers^9^. Our findings of an inverse relation between MoCA and PS suggest that BBB disruption is linked to brain function in the elderly population. However, the observed link between PS and cognition in our cohort was about equally large for the hippocampus, putamen, temporal WM and temporal cortex. When comparing hippocampal PS to the average PS over cortex, WM and basal ganglia, hippocampus showed the most pronounced MoCA associations; however, WM and basal ganglia showed almost equally large effects, and PS in all four regions were significantly related to MoCA when statistically controlling for age and sex. Further, Verheggen et al.^13^ and van de Haar et al.^14^ linked global PS in GM and WM to memory decline over 12 years^13^ and found global PS increases in AD patients^14^. In addition, other studies found no associations between hippocampal PS and cognition^10–12^. Collectively, the ambiguities in previous DCE studies and the only slightly larger effect for the hippocampus and temporal lobe compared to whole-brain averaged PS in our study suggest that cognitive decline due to BBB breakdown is mainly a whole-brain phenomenon. However, considering the discrepancies among existing cross-sectional studies, longitudinal population-based studies (where cohort effects are less of an issue) are needed to probe subtle BBB leakages as a central mechanism in aging and as an early trigger of cognitive decline.

In line with expectations, ${v}_{p}$ was positively related to tCBF for all regions. These findings are not surprising and suggest that a larger inflow of blood to the brain indicates a larger, or more dense, capillary network. Local flow rate–${v}_{p}$ associations were also found for territories supplied by the left ICA and left and right MCA, whereas non-significant trends were observed for the right ICA and the PCA-supplied regions. The more pronounced association for tCBF–${v}_{p}$ likely reflects that tCBF more accurately reflects tissue perfusion due to collateral circulation, and that individual measurements in the PCA are much more susceptible to errors. In contrast to our expectations, 4D flow MRI pulsatility was unrelated to BBB permeability for all regions. This was observed for ICA and distal arteries in relation to hippocampal, cortical, white matter, basal ganglia and a whole-brain averaged PS, but also when considering local relationships between pulsatility in the left and right ICA, MCA, and PCA and PS in the supplied brain region. Based on previous findings, we had reasons to believe that the hippocampal microvasculature would be particularly susceptible to pulsatile stress. Previous studies have linked arterial^41,44^ and CSF^41^ pulsatility to hippocampal volume^41^ and hippocampus-sensitive episodic memory^44^. However, our observations do not support the idea that excessive vascular pulsatility propagated to the microcirculation would trigger hippocampal capillary damage. Surprisingly, PS showed positive (non-significant) trends against tCBF for all regions. However, these trends were all suppressed when controlling PS for ${v}_{p}$. This highlights a potential physiological issue with the Patlak model when interpreting PS (or ${K}_{trans}$) as BBB permeability, although it actually reflects the product of permeability and vascular surface area^1^. Alternatively, diffusion prepared pseudo-continuous arterial spin labeling can be used to map the water exchange (${k}_{w}$) across the BBB^45^. In a recent comparison between ${k}_{w}$ and PS (or ${K}_{trans}$), non-significant correlations were found for most brain regions, highlighting how these methods reflect different physiological mechanisms^46^.

Regarding the fractional plasma volume (${v}_{p}$), positron emission tomography (PET) estimates suggest an age-related decline of the vascular volume fraction of approximately 0.5% per year for grey and white matter throughout the lifespan^47^. Our results indicate a relative decline for hippocampal ${v}_{p}$ of 1.97% per year in the ages 68 to 84, suggesting that the hippocampus could be particularly susceptible to age-related vascular changes in terms of reduced capillary density, something that should be further investigated in longitudinal population-based studies. Further, regional brain volumes were inversely related to age for 7/15 brain regions, to pulsatility for 1/15 brain regions, whereas volumes were unrelated to PS and MoCA. The age-volume associations were not surprising for an age where atrophy likely have set in, and the non-significant links for 8/15 regions likely reflect a narrow age span and a small cohort. However, the positive volume-pulsatility correlation for one brain region (frontal WM) contradicts previous findings of inverse relationships between regional brain volumes and pulsatility^41,48^, but should not be interpreted as significant when the 14 other regions showed no association.

There are some limitations in the current study. An important regulator of BBB permeability is ApoE4. Hippocampal BBB permeability predicts cognitive decline, specifically in ApoE4 + carriers^9^, and ApoE4 + individuals show marked increases in hippocampal BBB permeability when comparing both healthy and cognitively impaired individuals, seperately^10^. Hence, not including APOE4 status is a limitation. Another limitation concerns the pulsatility measurements. Artificial increases in carotid pulsatility seems to propagate to the microcirculation and trigger BBB breakdown and capillary loss^17^; however, these rodents experienced a pulse pressure increase of almost 200% following a surgical constriction of the aorta^17^. Previous studies have reported longitudinal increases in the cerebral arterial PI of approximately 1.2% per year in healthy older adults^49^. Hence, age-related pulsatility increases may be insufficient to trigger BBB breakdown. Further, the 4D flow measurements may suffer from partial volume errors (PVEs), especially in smaller vessels^50^. However, as previously shown using a phase contrast MRI simulation framework, PVEs induce a relative flow rate increase at each cardiac phase, leaving the PI relatively unaffacted^23^. Regarding the PS–cognition associations, we also note that our cognitive test battery was limited to the MoCA test, known to be sensitive to degeneration of the medial temporal lobe, including the hippocampus^51^. Hence, future studies on subtle BBB leakages should include more comprehensive test batteries designed to capture decline in other cognitive domains, such as working memory and processing speed. Finally, quantifying subtle BBB leakages is challenging compared to clinical DCE MRI applications. This is mainly due to the low contrast concentrations that cross the BBB in relatively healthy tissue^37^. In line with consensus recommendations^1,39^, we extracted our VIF in the SSS to minimize the impact of subject motion, partial volume errors, and inflow effects, a slow contrast injection to better resolve the concentration peak in the VIF, a simple averaging approach without voxel exclusion based on pre-defined leakage thresholds, and multiple linear regression to solve the Patlak equation for PS and ${v}_{p}$. In addition, our semi-automatic centerline approach for VIF extraction showed excellent interrater reliability for PS and ${v}_{p}$ in all the investigated brain regions. However, although a two-step approach was used to correct motion between DCE frames, residual head motion within frames could still lead to pseudo-random PS errors that affect all regions^1^ and is difficult to account for, something that could partially explain interregional correlations in PS.

In conclusion, BBB leakage rates (PS) and plasma volume fraction (${v}_{p}$) were estimated for a population-based cohort using DCE MRI, and evaluated in relation to age, cognition, and cerebral hemodynamics. For multiple brain regions, leakage rates were inversely related to cognition assessed by the MoCA score, with only slightly more pronounced associations for the hippocampus than for whole-brain averaged PS, suggesting the PS—cognition links mainly reflect a whole-brain phenomenon. However, PS was unrelated to age and cerebral 4D flow MRI hemodynamics, including pulsatility, providing no evidence of excessive vascular pulsatility as a trigger of BBB disruption under physiological conditions.

### Supplementary Information

Supplementary Information.

## Data Availability

The datasets generated and/or analyzed during the current study are not publicly available due to protection of data privacy of the study participants. Data is available from the corresponding authors open reasonable request.
