# Supplementary material for: Blood–brain barrier integrity is linked to cognitive function, but not to cerebral arterial pulsatility, among elderly
Source: Sci Rep. 2024 Jul 3;14:15338. doi: 10.1038/s41598-024-65944-y (PMC11222381; doi:10.1038/s41598-024-65944-y)
Supplement: Supplementary file 1 — Supplementary Information. [file 41598_2024_65944_MOESM1_ESM.pdf]

Supplementary information for:

# Blood-brain barrier integrity is linked to cognitive function, but not to cerebral arterial pulsatility, among elderly

Tomas Vikner[1,2,6,*], Anders Garpebring[1], Cecilia Björnfot[1], Lars Nyberg[1,3,4], Jan Malm[5], Anders Eklund[1,2,3], and Anders Wåhlin[1,2,3]

1. Department of Diagnostics and Intervention, Umeå University, S-90187 Umeå, Sweden
2. Department of Applied Physics and Electronics, Umeå University, S-90187 Umeå, Sweden
3. Umeå Center for Functional Brain Imaging (UFBI), Umeå University, S-90187 Umeå, Sweden
4. Department of Integrative Medical Biology (IMB), Umeå University, S-90187 Umeå, Sweden
5. Department of Clinical Science, Neurosciences, Umeå University, S-90187 Umeå, Sweden
6. Department of Medical Physics, School of Medicine and Public Health, University of Wisconsin-Madison, Madison, WI, 53792, USA

**Corresponding authors:**

Tomas Vikner (tomas.vikner@umu.se) +1 (608) 387-7438

Department of Radiation Sciences, Radiation Physics and Biomedical Engineering, Umeå University, SE 901 87 Umeå, Sweden

Anders Wåhlin (anders.wahlin@umu.se) +46 70 253 74 26

Department of Applied Physics and Electronics, Umeå University, SE 901 87 Umeå, Sweden

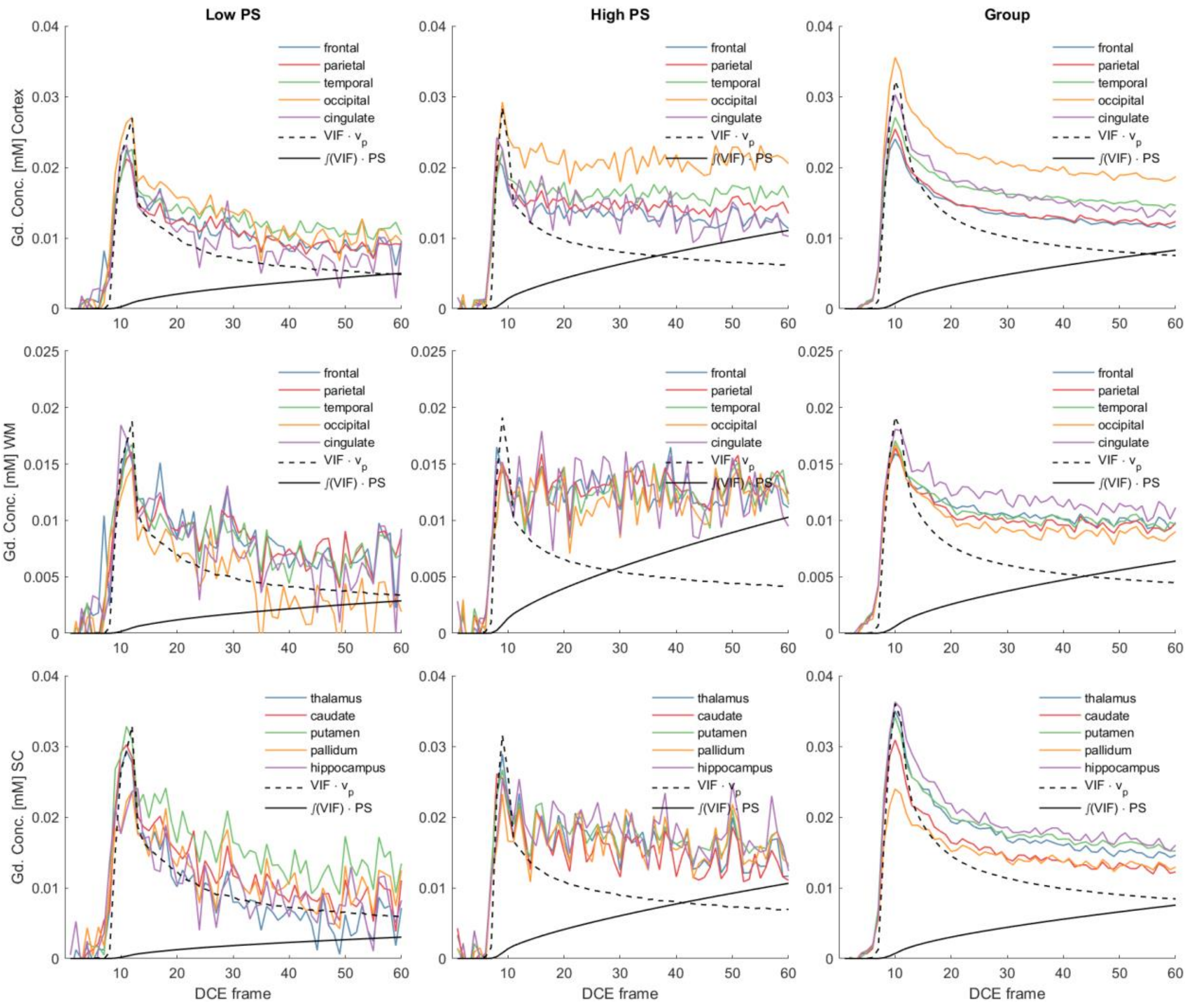


**Sup. Fig. 1.** Gadolinium (Gd) concentration curves [mM] for cortical, white matter (WM), and subcortical gray matter (SC) regions, for example individuals with low and high permeability-surface area product (PS) and for group-averaged concentrations. The vascular input function (VIF) was normalized through multiplication by the average of vascular fraction ($v_p$) within the corresponding (5x) subregions, such that an increasing gap over time between tissue-concentration and the VIF indicates accumulation (due to PS) over time. The $\int$ (VIF) $\cdot$ PS term reflects the contrast accumulation due to PS (averaged over the 5 ROIs of each plot), indicating high Gd accumulation over time in the high PS case.

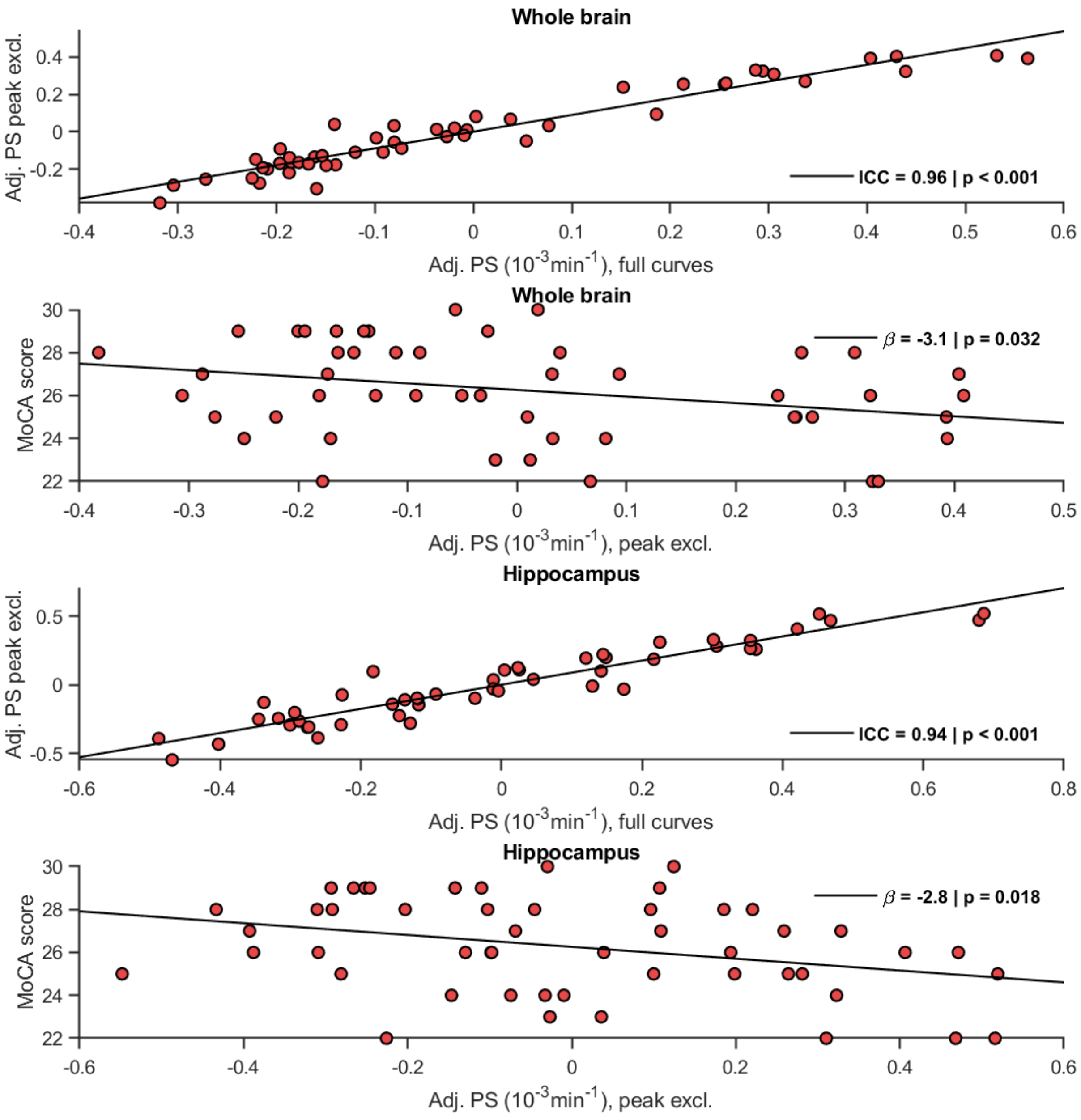


**Sup. Fig. 2.** Evaluation of permeability-surface area product (PS; adjusted for age and sex) when excluding the contrast injection peak before Patlak analysis. The vascular input function (VIF) peak frame was automatically detected and a start index for Patlak fit was selected as two frames after the peak frame. Intraclass correlation (ICC) was used to evaluate PS obtained from using the full concentration curves (as for the main results) against PS obtained by excluding the injection peak. The regression results (β, p) show that PS–cognition links (assessed by the Montreal Cognitive Assessment score, MoCA) links are still significant for the hippocampus and borderline-significant for whole-brain averaged PS after excluding early frames.

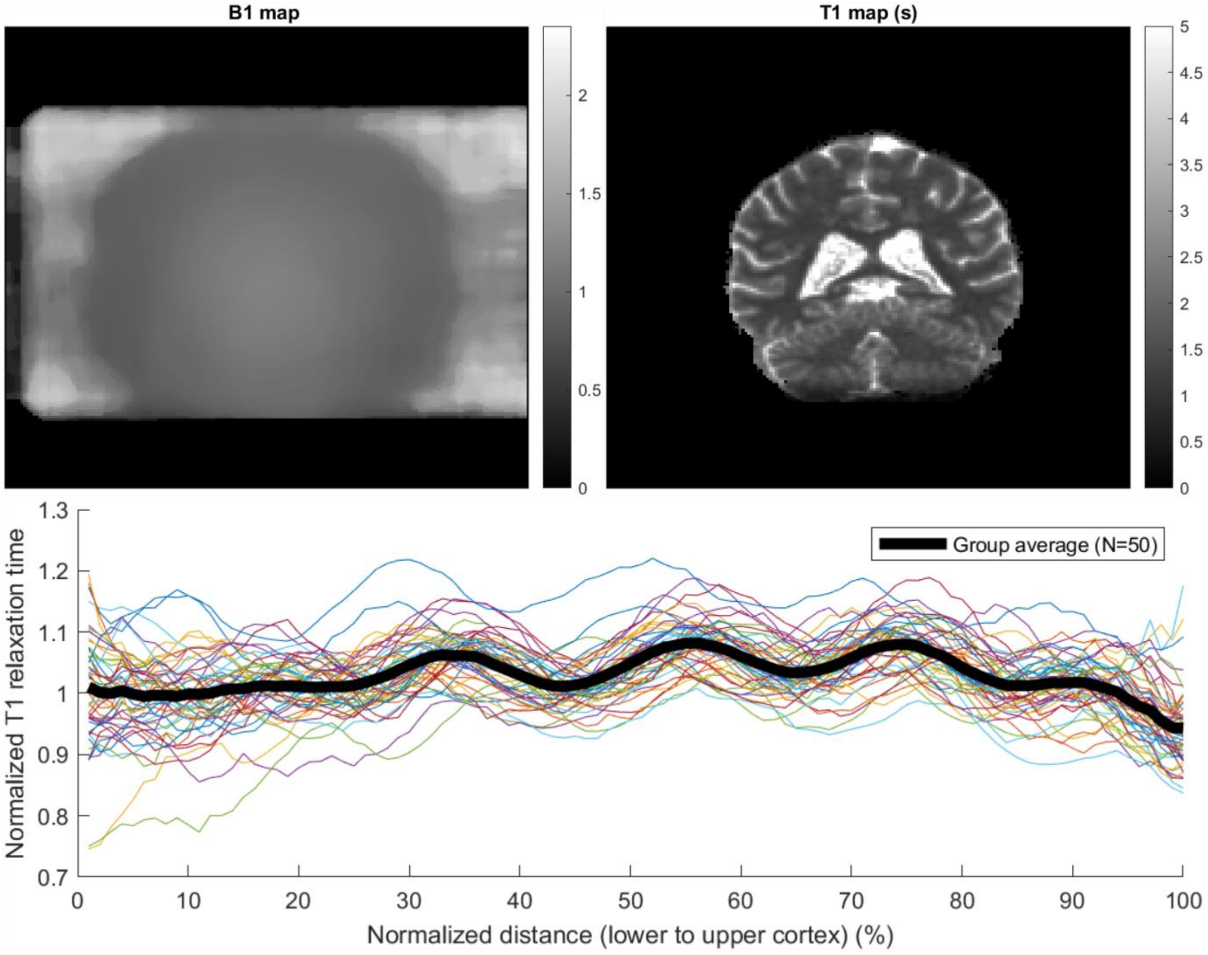


**Sup. Fig. 3.** B1 map and T1 relaxation time map for an example participant, and T1 relaxation time as a function of normalized distance in the axial direction for the cohort (N=50), indicating that the slab excitation profile may be non-uniform at the top of the cortex. Normalized distance was defined from 0% (inferior edge) to 100% (superior edge) of the cortex. The T1 relaxation time was defined by averaging over voxels slice by slice, and normalized such that 1 reflects the values in the middle half of the imaging slab.

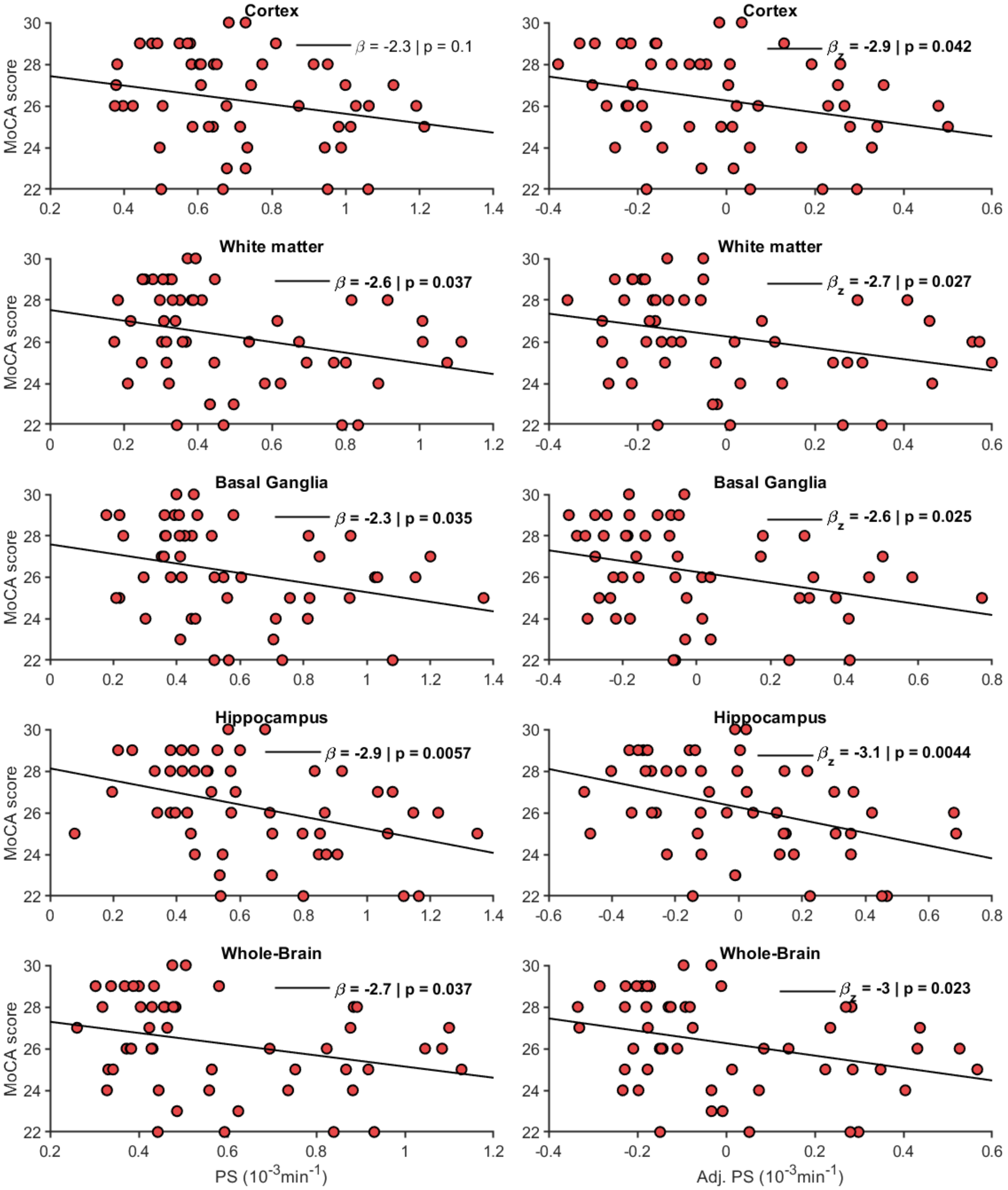


**Sup. Fig. 4.** Permeability-surface area product (PS; $10^{-3}min^{-1}$) in relation to the Montreal cognitive assessment (MoCA) score for cortex, white matter (WM), basal ganglia (BG) and hippocampus, after excluding frontal and parietal cortex (where the RF pulse slab excitation profile may be non-uniform) from the cortex region. Hence, cortex included temporal, occipital and cingulate regions; WM included frontal, parietal, temporal, occipital and cingulate regions; BG included caudate, putamen, and pallidum; and whole-brain PS was obtained by averaging PS over all (13) subregions. The adj. PS was adjusted for age and sex by regression. The β coefficients indicate change in MoCA score per 1.0 x $10^{-3}min^{-1}$ change in PS.

**Sup. Tab. 1.** Permeability-surface area product (PS) and fractional plasma volume ($v_p$) in relation to 4D flow MRI derived blood flow (BF) rates and pulsatility index (PI) with region-specific associations.

| | **Left PI-PS** | **Right PI-PS** | **Left BF-$v_p$** | **Right BF-$v_p$** |
|---|---|---|---|---|
| **Vessel–region** | β (CI) | β (CI) | β (CI) | β (CI) |
| ICA–Front. Ctx. | 0.2 (-0.1, 0.5) | -0.04 (-0.3, 0.3) | 2.2 (1.1, 3.3) *** | 0.4 (-0.9, 1.6) |
| ICA–Front. WM | 0.2 (-0.2, 0.6) | -0.07 (-0.5, 0.4) | 1.7 (0.7, 2.7) ** | 0.05 (-1.2, 1.3) |
| ICA–Hippocamp. | 0.06 (-0.4, 0.5) | -0.13 (-0.5, 0.3) | 4.1 (0.9, 7.2) * | 1.5 (-1.3, 1.3) |
| MCA–Temp. Ctx. | -0.05 (-0.3, 0.2) | -0.01 (-0.3, 0.3) | 2.9 (0.5, 5.4) * | 2.5 (0.3, 4.8) * |
| MCA–Temp. WM | -0.09 (-0.4, 0.2) | -0.09 (-0.5, 0.3) | 2.3 (0.6, 4.0) * | 2.2 (0.3, 4.0) * |
| MCA–Basal Gang. | -0.02 (-0.4, 0.3) | -0.10 (-0.5, 0.3) | 3.4 (1.1, 5.6) ** | 4.1 (1.5, 6.7) ** |
| PCA–Occ. Ctx. | -0.09 (-0.5, 0.3) | 0.25 (-0.1, 0.6) | 5.0 (-4.4, 14) | 6.8 (-1.0, 15) |
| PCA–Occ. WM | -0.10 (-0.4, 0.2) | 0.09 (-0.3, 0.5) | 2.1 (-1.2, 5.4) | 1.8 (-1.5, 5.1) |
| PCA–Hippocamp. | -0.11 (-0.4, 0.2) | -0.07 (-0.4, 0.3) | 7.1 (-2.7, 17) | 6.0 (-2.7, 15) |

**Note:** R and p-values obtained from Pearson correlation. Unadjusted p-values: *$p<0.05$; **$p<0.01$; ***$p<0.001$. The 4D flow hemodynamic parameters (PI and BF) and the microvascular DCE parameters (PS and $v_p$) were specifically evaluated for left vessel vs. left hemisphere and right vessel vs. right hemisphere by considering region-specific arterial supply. The β coefficients reflect change in PS ($10^{-3}min^{-1}$) and $v_p$ (%) per change in PI and BF rate (scaled to L/min).

**Sup. Tab. 2.** PS adjusted for $v_p$ in relation to age, tCBF, distal PI, and Montreal Cognitive Assessment (MoCA).

| | **Age** | **tCBF** | **Distal PI** | **MoCA** |
|---|---|---|---|---|
| **PS adj.** | β (CI) | β (CI) | β (CI) | β (CI) |
| Front. Ctx. | -0.01 (-0.02, 0.01) | 0.29 (-0.27, 0.84) | 0.058 (-0.28, 0.39) | -3.0 (-6.3, 0.25) |
| Pari. Ctx. | -0.001 (-0.02, 0.01) | 0.31 (-0.28, 0.90) | 0.076 (-0.26, 0.44) | -2.7 (-5.8, 0.42) |
| Temp. Ctx. | -0.004 (-0.02, 0.01) | 0.12 (-0.44, 0.68) | 0.072 (-0.26, 0.41) | -4.0 (-7.2, -0.77) * |
| Occ. Ctx. | -0.002 (-0.02, 0.02) | -0.05 (-0.85, 0.76) | 0.012 (-0.47, 0.49) | -2.2 (-4.5, 0.10) |
| Cing. Ctx. | -0.005 (-0.02, 0.01) | 0.01 (-0.69, 0.70) | 0.130 (-0.28, 0.54) | -0.34 (-3.1, 2.4) |
| Front. WM | -0.009 (-0.03, 0.01) | 0.13 (-0.60, 0.86) | 0.063 (-0.37, 0.50) | -2.1 (-4.6, 0.45) |
| Pari. WM | -0.003 (-0.02, 0.02) | 0.45 (-0.27, 1.20) | 0.083 (-0.36, 0.52) | -2.6 (-5.1, -0.10) * |
| Temp. WM | -0.004 (-0.02, 0.01) | 0.21 (-0.47, 0.90) | -0.019 (-0.43, 0.39) | -3.5 (-6.1, -0.91) ** |
| Occ. WM | -0.002 (-0.02, 0.02) | 0.23 (-0.55, 1.00) | -0.039 (-0.51, 0.43) | -2.6 (-4.9, -0.29) * |
| Cing. WM | -0.005 (-0.03, 0.02) | -0.09 (-0.96, 0.78) | 0.003 (-0.52, 0.52) | -0.99 (-3.2, 1.2) |
| Thalamus | -0.003 (-0.02, 0.02) | 0.06 (-0.76, 0.88) | -0.046 (-0.53, 0.44) | -1.6 (-3.9, 0.69) |
| Caudate | -0.007 (-0.03, 0.01) | 0.06 (-0.76, 0.87) | -0.039 (-0.52, 0.45) | -1.5 (-3.8, 0.81) |
| Putamen | -0.010 (-0.03, 0.01) | 0.18 (-0.56, 0.93) | 0.040 (-0.40, 0.49) | -3.1 (-5.5, -0.73) * |
| Pallidum | -0.013 (-0.04, 0.01) | 0.36 (-0.56, 1.30) | -0.045 (-0.60, 0.51) | -2.1 (-4.1, -0.15) * |
| Hippocampus | -0.001 (-0.02, 0.02) | 0.22 (-0.59, 1.00) | 0.024 (-0.46, 0.51) | -3.0 (-5.2, -0.86) ** |

**Note:** β and p-values obtained from linear regression. Unadjusted p-values: *$p<0.05$; **$p<0.01$. The β coefficients reflect change in PS ($10^{-3}min^{-1}$) in per change in age, total cerebral blood flow (tCBF; L/min) and distal PI, or change in MoCA score per change in PS ($10^{-3}min^{-1}$).

**Sup. Tab. 3.** Regional brain volumes (ml) in relation to age (years), permeability-surface area product (PS), distal arterial pulsatility index (PI), and Montreal cognitive assessment (MoCA) score.

| | **Age** | **PS ($10^{-6}min^{-1}$)** | **Distal PI** | **MoCA** |
|---|---|---|---|---|
| **Volume** | β (CI) | β (CI) | β (CI) | β (CI) |
| Front. Ctx. | -0.97 (-2.3, 0.37) | 5.7 (-23, 34) | 15 (-18, 48) | 0.64 (-1.8, 3.1) |
| Pari. Ctx. | -0.84 (-1.7, 0.0024) | 9.1 (-7.7, 26) | 6.4 (-15, 28) | 0.021 (-1.6, 1.6) |
| Temp. Ctx. | -0.68 (-1.3, -0.012) * | 7.5 (-6, 21) | 9.7 (-7.3, 27) | 0.47 (-0.8, 1.7) |
| Occ. Ctx. | -0.45 (-0.75, -0.15) ** | 3.5 (-0.49, 7.5) | 0.34 (-7.8, 8.5) | 0.0012 (-0.6, 0.6) |
| Cing. Ctx. | -0.015 (-0.15, 0.12) | -1.3 (-3.4, 0.73) | 1.0 (-2.4, 4.4) | -0.086 (-0.34, 0.17) |
| Front. WM | -0.83 (-1.3, -0.32) ** | 2.8 (-5.6, 11) | 17 (3.4, 30) * | -0.057 (-1.1, 0.97) |
| Pari. WM | -0.34 (-0.86, 0.17) | 1.9 (-6.2, 10) | 4.8 (-8, 18) | -0.55 (-1.5, 0.39) |
| Temp. WM | -0.3 (-0.56, -0.041) * | 0.35 (-4, 4.7) | 5.9 (-0.73, 12) | -0.13 (-0.64, 0.37) |
| Occ. WM | -0.33 (-0.59, -0.065) * | 1.6 (-2.4, 5.5) | 1.1 (-5.8, 8) | -0.055 (-0.56, 0.45) |
| Cing. WM | -0.083 (-0.2, 0.033) | 0.068 (-1.3, 1.5) | 0.015 (-2.9, 3) | -0.25 (-0.45, -0.045) |
| Thalamus | -0.06 (-0.11, -0.013) * | 0.41 (-0.23, 1) | 0.5 (-0.73, 1.7) | 0.019 (-0.073, 0.11) |
| Caudate | -0.035 (-0.089, 0.02) | 0.37 (-0.38, 1.1) | 0.92 (-0.43, 2.3) | -0.024 (-0.13, 0.076) |
| Putamen | -0.027 (-0.1, 0.048) | 0.43 (-0.74, 1.6) | 0.29 (-1.6, 2.2) | -0.047 (-0.18, 0.09) |
| Pallidum | -0.022 (-0.05, 0.0054) | 0.15 (-0.19, 0.49) | 0.36 (-0.34, 1.1) | 0.02 (-0.032, 0.072) |
| Hippocampus | -0.042 (-0.07, -0.02) ** | 0.17 (-0.23, 0.57) | 0.28 (-0.43, 1) | -0.011 (-0.064, 0.042) |

**Note:** β and p-values obtained from linear regression. Unadjusted p-values: $*p<0.05$; $**p<0.01$. Regional volumes (ml) were obtained from FreeSurfer segmentations, normalized by adjusting for total intracranial volume through residualization by regression. The β coefficients reflect change in volume (ml) per change in age (years), PS ($10^{-3}min^{-1}$), distal PI and MoCA.
